# USING SOCIAL MEDIA POSTS TO UNDERSTAND THE HEALTH INFORMATION NEEDS FOR OLDER ADULTS WITH DEMENTIA

**DOI:** 10.1093/geroni/igae098.4341

**Published:** 2024-12-31

**Authors:** Ebenezer Larnyo, Karim Benyassine, Natasha Preece

**Affiliations:** University of California, Santa Barbara, Santa Barbara, California, United States; University of California, Santa Barbara, Santa Barbara, California, United States; University of California, Santa Barbara, Santa Barbara, California, United States

## Abstract

Older adults with dementia and their caregivers face significant challenges, necessitating innovative approaches to support their evolving health information needs. This study leverages the power of social media to explore these needs, analyzing 664 posts from Reddit communities dedicated to dementia and Alzheimer’s disease. Thematic analysis and sentiment analysis were employed to identify key themes and emotional undertones. Additionally, deep learning models (convolutional neural network (CNN), transformers, bidirectional long short-term memory (BI-LSTM), and gated recurrent unit (GRU)) were compared for sentiment prediction accuracy, with CNN demonstrating superior performance. Our findings reveal that caregivers constitute the majority of contributors, sharing experiences related to caregiving burden, dementia symptoms, and emotional challenges. Despite the inherent difficulties, a predominantly positive sentiment prevailed, highlighting the importance of social connection and support. This research underscores the potential of social media as a valuable resource for understanding the experiences of dementia caregivers. By harnessing these insights, healthcare providers and policymakers can develop more effective interventions and support systems to improve the quality of life for both patients and caregivers.

